# Clustering of Multiple Risk Behaviors Among a Sample of 18-Year-Old Australians and Associations With Mental Health Outcomes: A Latent Class Analysis

**DOI:** 10.3389/fpubh.2018.00135

**Published:** 2018-05-07

**Authors:** Katrina E. Champion, Marius Mather, Bonnie Spring, Frances Kay-Lambkin, Maree Teesson, Nicola C. Newton

**Affiliations:** ^1^NHMRC Centre of Research Excellence in Mental Health and Substance Use, National Drug and Alcohol Research Centre, University of New South Wales, Sydney, NSW, Australia; ^2^Department of Preventive Medicine, Feinberg School of Medicine, Northwestern University, Chicago, IL, United States; ^3^Priority Research Centre for Brain and Mental Health, The University of Newcastle, Newcastle, NSW, Australia

**Keywords:** clustering, risk behavior, emerging adulthood, chronic disease, mental health

## Abstract

**Introduction:**

Risk behaviors commonly co-occur, typically emerge in adolescence, and become entrenched by adulthood. This study investigated the clustering of established (physical inactivity, diet, smoking, and alcohol use) and emerging (sedentary behavior and sleep) chronic disease risk factors among young Australian adults, and examined how clusters relate to mental health.

**Methods:**

The sample was derived from the long-term follow-up of a cohort of Australians. Participants were initially recruited at school as part of a cluster randomized controlled trial. A total of 853 participants (M_age_ = 18.88 years, SD = 0.42) completed an online self-report survey as part of the 5-year follow-up for the RCT. The survey assessed six behaviors (binge drinking and smoking in the past 6 months, moderate-to-vigorous physical activity/week, sitting time/day, fruit and vegetable intake/day, and sleep duration/night). Each behavior was represented by a dichotomous variable reflecting adherence to national guidelines. Exploratory analyses were conducted. Clusters were identified using latent class analysis.

**Results:**

Three classes emerged: “moderate risk” (moderately likely to binge drink and not eat enough fruit, high probability of insufficient vegetable intake; Class 1, 52%); “inactive, non-smokers” (high probabilities of not meeting guidelines for physical activity, sitting time and fruit/vegetable consumption, very low probability of smoking; Class 2, 24%), and “smokers and binge drinkers” (high rates of smoking and binge drinking, poor fruit/vegetable intake; Class 3, 24%). There were significant differences between the classes in terms of psychological distress (*p* = 0.003), depression (*p* < 0.001), and anxiety (*p* = 0.003). Specifically, Class 3 (“smokers and binge drinkers”) showed higher levels of distress, depression, and anxiety than Class 1 (“moderate risk”), while Class 2 (“inactive, non-smokers”) had greater depression than the “moderate risk” group.

**Discussion:**

Results indicate that risk behaviors are prevalent and clustered in 18-year old Australians. Mental health symptoms were significantly greater among the two classes that were characterized by high probabilities of engaging in multiple risk behaviors (Classes 2 and 3). An examination of the clustering of lifestyle risk behaviors is important to guide the development of preventive interventions. Our findings reinforce the importance of delivering multiple health interventions to reduce disease risk and improve mental well-being.

## Introduction

Chronic diseases, such as cardiovascular disease, diabetes, and cancers, are the leading cause of death in Australia (1) and worldwide (2). Physical inactivity, poor diet, smoking, and alcohol use are consistently identified as major behavioral risk factors for chronic disease (3–5). More recently, sedentary behavior and poor sleep have also emerged as key risk factors for poor health. For example, sitting time has been associated with an increased risk of all-cause, cardiovascular-, and cancer-related mortality (6, 7). Furthermore, short and long sleep duration has been shown to predict a greater risk of developing cardiovascular disease, coronary heart disease, and stroke (8, 9).

Emerging adulthood, typically defined as 18–25 (10, 11), is a critical developmental period when young people have increased exposure to risk behaviors, such as alcohol use and smoking (12, 13), while also acquiring greater autonomy over their food and lifestyle choices (14). Alarmingly, population data indicates that 95% of 18–24-year olds in Australia do not eat the recommended amount of vegetables, 59% do not eat the recommended amount of fruit, 47% do not meet guidelines for physical activity, over two-thirds drink alcohol at risky levels, 36% are overweight or obese, 29% are sedentary, and 16% are current smokers (15). Sleep disturbance is the fourth most common mental health problem for Australians aged between 12 and 24, after depression, anxiety and substance use, with the greatest sleep deprivation reported for the age group 20–24 years (16). In addition to being highly prevalent, these lifestyles risk behaviors commonly co-occur as clusters, as people engage in multiple risk behaviors concurrently (13). Risk behaviors typically have a synergistic effect, such that the co-occurrence of multiple risk behaviors increases the risk of chronic disease incidence and mortality, more so than the additive effects of single behaviors (8, 17–19).

The clustering of multiple risk behaviors is an area of growing research, with the majority of existing studies focusing on general adult populations (20). Although less studied, research focusing on emerging adults has been conducted, most notably in college or university student populations (21–26). Previous research has found distinct risk clusters, characterized by engagement in different risk behaviors and varying prevalence, and consistently concludes that risk behaviors, such as diet, physical inactivity, and substance use, co-occur in young adults.

Risk behaviors acquired or maintained during emerging adulthood often track into later adult life, influencing future adult health. For example, risk factor profiles in young adulthood have been shown to be strongly predictive of long-term coronary heart disease risk (27), whilst the maintenance of a healthy lifestyle throughout young adulthood is strongly associated with a low-cardiovascular disease risk profile in middle age (28). In addition to impacting future risk for physical disease, multiple health risk behaviors have been found to be associated with mental health problems among young adults. Specifically, young adults engaged in multiple lifestyle risk behaviors have been shown to exhibit higher rates of depression, anxiety, and distress than their counterparts who engage in fewer risk behaviors (22, 23, 29). Taken together, it is clear that prevention and early intervention strategies are needed among young people to reduce the risk of chronic physical diseases and mental disorders.

To date, there has been limited research examining the co-occurrence of multiple risk behaviors among emerging adults, especially in Australia (30), despite the high prevalence of lifestyle risk behaviors in this population (15). Furthermore, most studies examining risk clusters have not included sleep in their analyses. Additionally, sedentary behavior is less studied compared with traditional risk behaviors, such as diet, physical activity, and substance use. An investigation of the clustering of traditional and emerging risk behaviors among young Australians could provide critical information to guide the development and tailoring of future interventions. Also, given that one in four Australians aged 16–24 years experiences a mental disorder in any given year (31), an examination of how lifestyle risk behaviors relate to mental health outcomes is critical for informing holistic prevention approaches to improve both the physical and mental wellbeing of young people. An examination of the clustering of risk behaviors specifically among 18-year olds is important, as this age marks a transitional period where many young people transition out of school and into employment or further study. As adolescents transition out of school they are presented with unique challenges as it is a time where they acquire greater autonomy over their lifestyle choices (14), and are legally able to purchase alcohol and tobacco for the first time. As such, the aims of the present study were to conduct exploratory analyses to:

(i) Investigate the presence of clustering of six key risk behaviors—binge drinking, smoking, sleep duration, physical inactivity, fruit and vegetable intake, and sitting time—in a sample of 18-year-olds in Australia.
(ii) Determine whether the identified latent classes are associated with mental health outcomes and socio-demographic factors.

## Materials and Methods

### Participants

The sample was derived from the long-term follow-up of a cohort of young Australians. A total of 2,190 Year 8 students (13–14 year-olds) from 26 Australian secondary schools were recruited to a cluster randomized controlled trial of a substance use prevention program in 2012. Participants completed baseline assessments and a further four assessments between 2012 and 2015 representing post-test, 1-, 2-, and 3-year follow-ups [full details of the RCT are published elsewhere (32, 33)]. A 5-year follow-up of the cohort commenced in 2017, the first time participants were assessed since completing secondary school. The present study utilizes cross-sectional data collected from the 853 participants (mean age = 18.88 years, SD 0.42) who had completed the 5-year follow-up assessment at the time of the analysis.

### Procedure

Using multiple sources of locator information provided during previous assessments (including email, phone number, postal address, Facebook handle, and parents’ email address), participants were contacted and invited to complete the 5-year follow-up assessment. They were asked to complete an online self-report survey via the study website. Unlike previous waves of data collection, participants were no longer attending secondary school and thus completed the survey remotely at a location of their choice. This study was approved by the UNSW Sydney Human Research Ethics Committee (HC16881). All subjects gave written informed consent in accordance with the Declaration of Helsinki. Respondents were given the opportunity to receive $30 (or an equivalent voucher) as reimbursement for their time.

### Measures

Socio-demographic variables assessed were gender (male, female, and other), employment status (full-time, part-time, or unemployed), and current/completed tertiary education (none, trade/technical, and university/college). Self-reported body mass index (BMI) was calculated as weight (kilograms) divided by height (metres squared). Cut-offs from World Health Organization recommendations (34) were used to classify BMI as underweight (BMI < 18.5), normal range (BMI between 18.5 and 25), overweight (BMI > 25), or obese (BMI > 30). Consistent with prior research examining the clustering of multiple health behaviors (22, 25), engagement in each of the six risk behaviors was represented by a dichotomous variable reflecting adherence to national guidelines (0 = adherence to guideline, 1 = failing to meet guideline; see Table 1).

**Table 1:** Coding of risk behavior variables.

Risk behavior	Coding
Binge drinking in the past 6 months	1 = 5 or more standard drinks on one occasion, at least monthly
Tobacco use in the past 6 months	1 = smoked tobacco more than once in the past 6 months
Physical activity/week	1 = <150 min moderate activity, <75 min vigorous activity, or an equivalent combination/week
Fruit and vegetable consumption/day	Vegetables: 1 = <4–5 serves/day Fruit: 1 = <2 serves/day
Sleep duration/day	1 = <6 hours or >11 hours/night
Sitting time/day	1 = sitting 8 or more hours/day

0 = adherence to guidelines, “not at-risk,” 1 = failing to adhere to guidelines, “at-risk.”

#### Binge Drinking

A single item was used to assess binge drinking: “How often did you have five or more standard alcoholic drinks on one occasion in the past 6 months?” This measure is based on the Australian National Health and Medical Research Council guidelines and reflects international definitions of binge drinking (35). Responses were made on a six-point scale ranging from “never” to “daily or almost daily.” Responses were dichotomized so that binging monthly or more frequently was coded 1, and “never” or “less than monthly” coded as 0.

#### Tobacco Use

Smoking was assessed via the following item: “How often have you tried tobacco (cigarettes) in the last 6 months?” Response options were “none,” “once,” “tried more than once and less than five times,” or “tried more than five times.” Responses were binary coded so that using tobacco once or less in the past 6 months was coded 0, and using tobacco more than once was coded as 1.

#### Fruit and Vegetable Consumption

Fruit and vegetable intake was assessed using validated short items commonly used in health research (36, 37). Fruit intake was assessed via a single item: “About how many serves of fruit do you usually have each day?” Possible response options were “don’t eat fruit” “one serve or less,” “two to three serves,” “four to five serves,” “six serves or more.” Similarly, vegetable consumption was assessed by the following item: “About how many serves of vegetables do you usually eat each day?” with responses made on a comparable scale to fruit intake. Participants were provided with written information about what constitutes one serve of fruit/vegetables. In line with national dietary guidelines (38), responses were dichotomized so that poor fruit intake was defined as less than two serves/day, and insufficient vegetable intake classified as less than four to five serves/day.

#### Physical Activity

To calculate self-reported moderate-to-vigorous physical activity, participants completed four items from the International Physical Activity Questionnaire-Short Form (IPAQ). The IPAQ has demonstrated good psychometric properties in a diverse range of samples (39). Respondents were asked to indicate how many days during the past 7 days, and for how long each day (in hours and minutes), they had performed vigorous physical activities (like heavy lifting, digging, aerobics, or fast bicycling). Participants were also asked to report the number of days they did moderate physical activities (like carrying light loads, bicycling at a regular pace, or doubles tennis, excluding walking) in the past 7 days and how much time they usually spent doing these activities on one of those days (in hours and minutes). Consistent with guidelines for Australian adults (40), insufficient physical activity was defined as less than 150 minutes of moderate activity, less than 75 minutes of vigorous activity, or an equivalent combination (e.g., 100 minutes of moderate activity and 25 minutes of vigorous activity would be classified as sufficient, representing two-thirds, and one-third of the total requirement, respectively).

#### Sedentary Behavior

Daily sitting time (in hours) was assessed using a single item from the IPAQ (39): “How many hours do you spend sitting in a typical 24-hour day (e.g., traveling to/from school, university or work; at school, university or work; watching television, using a computer at home and leisure time)?” There are currently no guidelines for Australian adults in regard to sitting time, however, based on previous Australian research (8, 41), sitting for eight or more hours per day was coded as “at-risk” in the present study.

#### Sleep Duration

To assess sleep duration (in hours), respondents were asked “How many hours in each 24-hour day do you usually spend sleeping (including at night and naps).” In line with sleep recommendations for young adults aged 18–25 years (42), insufficient sleep duration was defined as less than 6, or more than 11, hours per night.

#### Psychological Distress

The Kessler 6 (K6) (43) is a six-item scale used to measure psychological distress. For each item, participants were asked to rate how often they had felt a specific kind of distress in the past 4 weeks (“0 = none of the time” to “4 = all of the time”). Scores were summed to produce a total score (possible range: 0–24), with higher scores representing greater distress. The K6 has been found to have very good concordance with independent clinical ratings of psychological distress (44) and demonstrated good internal consistency in this study (α = 0.89).

#### Symptoms of Anxiety and Depression

The Brief Symptoms Inventory (BSI) (45) was used to assess symptoms of anxiety and depression, using the BSI Anxiety and Depression subscales, respectively. Both subscales showed good to excellent internal consistency, with α = 0.92 for the Depression scale and α = 0.89 for Anxiety. Participants were asked to report how much they had experienced symptoms of anxiety (e.g., “nervous or shakiness inside”) and depression (e.g., “feeling lonely”) and in the past 6 months on a five-point scale (0 = not at all to 4 = often). Scores were summed separately for the depression and anxiety subscales, yielding total scores between 0 and 24, with higher scores indicating greater symptoms.

### Statistical Analysis

Descriptive statistics were generated in R, version 3.4.2 (46). Data were cleaned to identify invalid responses on the outcome variables. Responses were coded as missing when participants entered an invalid value for an item (e.g., hours greater than 24 h/day). Where participants gave a range (e.g., “7–8 h”) instead of a finite numeric response, data were converted to the midpoint (e.g., to 7.5 h). Attrition analyses were conducted to compare participants who completed the 5-year follow-up assessment, to those who did not, in terms of gender, baseline drinking, and baseline binge drinking. Latent class analysis (LCA) (47) was used to identify clusters of behaviors among the participants, based on indicators of lifestyle risk. LCA models use patterns of responses on observed categorical variables to classify individuals into latent classes, where in each class there are different response probabilities across items. The poLCA package for R (version 1.4.1) was used to fit the latent class models (48). To select the number of clusters that best fit the data, we first fit a two-class model and successively increased the number of classes by one, up to a six-class model. Models were compared using the sample-size adjusted Bayesian information criterion (aBIC), Akaike information criterion (AIC), BIC, and relative entropy, which are widely accepted for LCA methods (49). The best model was selected on the basis of these fit statistics (with lower aBIC, AIC, and BIC indicating better fit), but also based on the interpretability of the estimated clusters. To ensure that the global maximum likelihood solution was found, each model was estimated 50 times with random initial parameters and the iteration with the lowest log-likelihood was selected as the final model.

Associations between class membership and mental health and socio-demographic factors were examined using χ^2^ tests (for categorical factors) and ANOVA analyses (for continuous measures). To account for uncertainty in class assignments, we repeated these analyses using pseudo-class draws, sampling from each participant’s posterior probability of class assignment (50). We generated 100 sets of pseudo-class draws and repeated the original comparisons using these predicted classes, combining the results of the sets using rules for multiple imputation (51). When ANOVA analyses revealed significant differences between classes, pairwise comparisons were conducted using the Tukey method to adjust for multiple comparisons. When examining associations between gender and the latent classes, participants who identified as “other” were excluded from comparisons due to low cell counts (*n* = 4).

## Results

### Sample Characteristics

A total of 853 participants (52.6% male; mean age = 18.8 years, SD = 0.42) completed the online survey. Table 2 summarizes the full sample characteristics. Results from the attrition analyses indicated that participants who did not complete the 5-year follow-up survey were more likely to be male and had significantly higher rates of binge drinking at baseline (see Table 3).

**Table 2:** Sample characteristics (*n* = 853).

	Male	Female	Other
Current education [*n* (%)]			
None	54 (12.0)	46 (11.5)	–
Trade/technical	41 (9.1)	9 (2.2)	–
University/college	354 (78.8)	345 (86.2)	4 (100.0)

Current employment [*n* (%)]			
Full-time employed	50 (11.1)	22 (5.5)	–
Part-time/casual employed	292 (65.0)	303 (75.8)	2 (50.0)
Unemployed	101 (22.5)	72 (18.0)	2 (50.0)
Other	6 (1.3)	3 (0.8)	–

Body mass index [M (SD)]	23.33 (4.68)	22.30 (3.47)	25.35 (2.40)
Category [*n* (%)]			
Underweight	20 (4.5)	24 (6.0)	–
Normal range	333 (74.2)	310 (77.5)	2 (50.0)
Overweight	62 (13.8)	49 (12.2)	2 (50.0)
Obese	28 (6.2)	13 (3.2)	–

Psychological distress [M (SD)]	5.54 (4.98)	6.65 (4.88)	15.00 (8.04)
BSI anxiety [M (SD)]	3.40 (4.39)	5.05 (5.00)	7.75 (5.74)
BSI depression [M (SD)]	5.21 (5.33)	6.84 (5.47)	8.75 (8.77)

**Table 3:** Comparison of participants in the current sample compared with those who did not complete the 5-year follow-up assessment.

	Current sample (*n* = 853)	Did not complete 5-year assessment (*n* = 1,337)	χ^2^ (1)	*p*-Value
Full standard drink (%)	15.2	18.3	3.50	0.062
Binge drinking (%)	2.7	5.0	6.69	0.010
Male (%)	52.6	59.6	9.66	0.002

### Prevalence of Lifestyle Risk Behaviors

Prevalence estimates for each risk behavior are shown in Table 4. Overall, over three quarters of the sample had insufficient intake of vegetables (80.2%) and more than half reported binge drinking at least monthly (52.4%). More than 40% showed inadequate consumption of fruit (41.9%), approximately one-third reported sitting for longer than recommended periods (32.5%), and approximately one quarter reported smoking (28.8%) or failing to meet physical activity guidelines (22.8%). Only a small minority (3.9%) got too little or too much sleep.

**Table 4:** Prevalence of lifestyle risk behaviors by gender.

	Male (*n* = 449)		Female (*n* = 400)		Other (*n* = 4)		Total (*n* = 853)	
Behavior	*N*	%	*N*	%	*N*	%	*N*	%
Binge drinking	247	55.0	198	49.5	2	50.0	447	52.4
Insufficient fruit intake	216	48.1	140	35.0	1	25.0	357	41.9
Insufficient veg intake	368	82.0	315	78.8	1	25.0	684	80.2
Physical inactivity	82	18.5	111	27.8	0	0.0	193	22.8
Sitting time	142	31.8	131	33.2	2	50.0	275	32.5
Insufficient sleep	17	3.8	16	4.0	0	0.0	33	3.9
Smoking	130	29.0	115	28.8	1	25.0	246	28.8

### Latent Class Analysis

**Figure 1:**
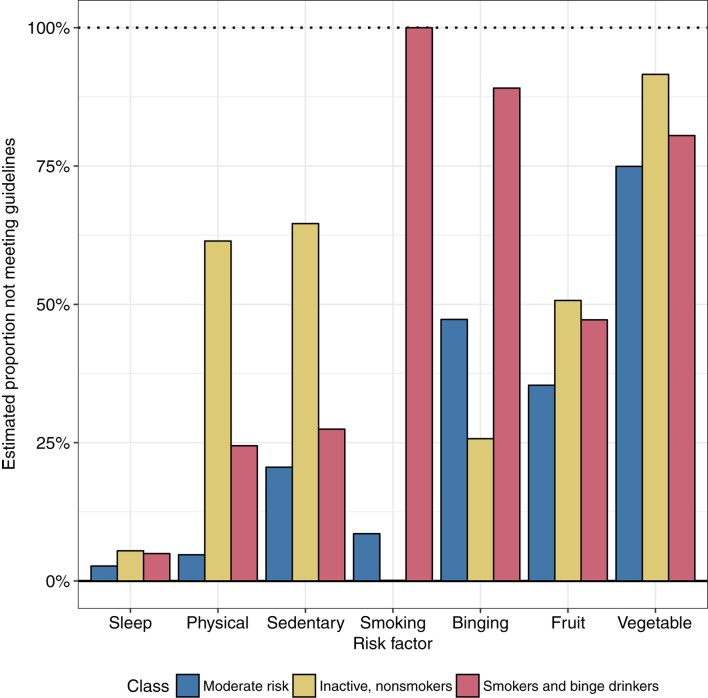
Estimated response probabilities of lifestyle risk behaviors in each latent class.

Model fit statistics from each model are shown in Table 5. The best fitting model, based on the model fit statistics and interpretability of the classes, was a three-class solution. The response probabilities of the risk behaviors within each of these three classes are shown in Figure 1. The average posterior probability of assignment to the most likely class was high for all classes (Class 1 84.9%, Class 2 83.0%, Class 3 87.7%), indicating good classification quality based on the threshold of 0.7 suggested by Nagin (52). The largest class, *Class 1*, had an estimated population proportion of 52.2% and was labeled “moderate risk.” This class had the lowest probability of engaging in most of the risk behaviors, with the exception of smoking and binge drinking, across the three classes. This class was characterized by a high probability of not eating enough vegetables and a moderate probability of binge drinking and poor fruit consumption. Participants in this group were highly likely to adhere to recommendations for sleep, physical activity, and sitting time, and were unlikely to smoke. The remainder of participants fell into two similarly sized classes. *Class 2* (23.5%) was labeled “inactive, non-smokers.” Participants in this class were likely to be non-smokers and reported the lowest probabilities of binge drinking than the other two classes. However, participants in this class were unlikely to achieve the recommended amounts of physical activity, sitting time, and serves of fruit and vegetables. *Class 3* (“smokers and binge drinkers,” 24.4%), was differentiated from the other classes by a high probability of smoking (100%) and of binge drinking. Participants in this class were also likely to fail to consume sufficient serves of fruit and vegetables per day.

**Table 5:** Model fit statistics for each of the fitted latent class analysis models.

Statistic	1 class	2 classes	3 classes	4 classes	5 classes	6 classes
Log-likelihood	−3,234.1	−3,153.4	−3,121.6	*−*3,107.4	−3,097.6	−3,092.4
aBIC	6,493.2	6,360.3	6,325.4	6,325.6	6,334.6	6,352.8
AIC	6,482.1	6,336.7	6,289.3	6,276.8	6,273.2	6,278.9
BIC	6,515.4	6,408.0	6,398.5	6,424.0	6,458.4	6,502.1
Relative entropy	N/A	0.62	0.68	0.74	0.65	0.70
df	7	15	23	31	39	47
G^2^	330.8	179.2	118.5	91.3	72.4	62.2
*p*(G^2^)	<0.001	<0.001	<0.001	<0.001	<0.001	0.07

#### Associations of Latent Class With Socio-Demographic Factors and Mental Health Outcomes

Means and percentages of socio-demographic factors and mental health outcomes are shown in Table 6. The three classes differed significantly in the proportion of males and females, with Class 2 (“inactive, non-smokers”) having a lower proportion of males, and employment, with a lower proportion of participants employed in Class 2. The classes did not differ significantly in the proportion of participants currently in tertiary education or BMI. Examination of mental health outcomes showed that the classes differed significantly in their levels of psychological distress, depression, and anxiety. Pairwise contrasts investigating these differences are shown in Table 7. Class 3 (“smokers and binge drinkers”) had significantly higher levels of psychological distress, anxiety, and depression compared with Class 1 (“moderate risk”). Class 2 (“inactive, non-smokers”) also had significantly higher levels of depression symptoms compared with Class 1 (“moderate risk”). As shown in Table 8, additional analyses using pseudo-class draws to account for the uncertainty in class assignments found a similar pattern of results, although there was no longer a significant difference in gender across the classes.

**Table 6:** Socio-demographic factors and mental health outcomes within each class.

	Class 1: “moderate risk” (*n* = 458)	Class 2: “inactive, non-smokers” (*n* = 166)	Class 3: “Smokers and binge drinkers” (*n* = 229)	Comparison	*p*-Value
Male (%)	55.2	44.6	53.3	χ^2^ (2): 6.08	**0.048**
In tertiary education (%)	90.0	94.6	88.2	χ^2^ (2): 4.70	0.095
Employed (%)	79.3	66.9	85.2	χ^2^ (2): 19.4	**<0.001**
BMI [mean (SD)]	23.10 (4.71)	22.43 (3.95)	22.67 (3.05)	*F*(2, 840): 1.82	0.16
Underweight (%)	5.7	6.8	3.1	–	–
Normal range (%)	74.2	75.9	81.6	–	–
Overweight (%)	14.6	11.7	12.3	–	–
Obese (%)	5.5	5.6	3.1	–	–
Psychological distress [mean (SD)]	5.63 (4.88)	6.23 (4.79)	6.97 (5.31)	*F*(2, 850): 5.59	**0.003**
BSI depression [mean (SD)]	5.33 (4.97)	6.58 (5.57)	6.89 (6.15)	*F*(2, 850): 7.50	**<0.001**
BSI anxiety [mean (SD)]	3.70 (4.26)	4.52 (4.87)	4.95 (5.48)	*F*(2, 850): 5.87	**0.003**

*Bolded p-values indicate p < 0.05*.

**Table 7:** Pairwise comparisons of socio-demographic factors and mental health outcomes across classes.

	Classes 1 vs 2		Classes 1 vs 3		Classes 2 vs 3	
	χ^2^ (1)	*p*-Value	χ^2^ (1)	*p*-Value	χ^2^ (1)	*p*-Value
Gender	5.62	**0.018**	0.28	0.60	2.57	0.11
Employment	9.58	**0.002**	3.10	0.078	17.40	**<0.001**

	***t*(850)**	***p*-Value**	***t*(850)**	***p*-Value**	***t*(850)**	***p*-Value**

Psychological distress	−1.33	0.38	−3.32	**0.003**	−1.46	0.31
BSI depression	−2.54	**0.030**	−3.55	**0.001**	−0.56	0.084
BSI anxiety	−1.93	0.13	−3.28	**0.003**	−0.89	0.65

*Bolded p-values indicate p < 0.05*.

**Table 8:** Pooled estimates from pseudo-class analyses where class assignments were sampled 100 times from the posterior class probabilities.

	*D*	*p*-Value	df
Gender	0.56	0.570	2, 987.4
In tertiary education	1.69	0.185	2, 2,032.9
Employed	6.04	**0.002**	2, 1,818.5
BMI	0.78	0.461	2, 937.5
Psychological distress	6.56	**0.002**	2, 556.5
BSI depression	8.32	**<0.001**	2, 689.2
BSI anxiety	5.30	**0.005**	2, 535.1

*Bolded p-values indicate p < 0.05. The test statistic D is approximately F-distributed, with degrees of freedom adjusted for the variance of the individual estimates*.

## Discussion

This study examined the clustering of six key risk behaviors—binge drinking, smoking, physical inactivity, sitting time, poor sleep, and fruit/vegetable consumption—among a sample of 18-year olds in Australia. It makes an important contribution to the literature by examining *traditional* chronic disease risk behaviors (diet, smoking, alcohol use, and physical inactivity) in combination with *emerging* risk behaviors (such as sleep and sedentary behavior) (8) during an important transitional life stage, characterized by change, uncertainty (11) and increased independence. Regardless of class membership, the present study found a high prevalence of risk behaviors among the sample, most notably insufficient vegetable consumption (80.2%), binge drinking (52.4%), and inadequate consumption of fruit (41.9%). These findings are largely consistent with recent national survey data in Australia, which found that 95% of 18- to 24-year olds did not consume the recommended amount of vegetables, 59% do not eat recommended amount of fruit, and over two-thirds drink alcohol at risky levels (15). In light of strong evidence that an increased consumption of vegetables and fruit can reduce the risk of many chronic diseases, including hypertension, coronary heart disease and stroke, and all-cause mortality (53), education about the importance of meeting dietary recommendations is urgently needed (54). On the other hand, nearly all participants reported getting adequate sleep (6–11 h/day), which is encouraging given that inadequate sleep duration has been associated with health problems such as obesity, cardiovascular disease, and mortality (9, 55–57). This finding is in contrast to recent prevalence estimates in the United States in which 23% of 18- to 24-year-old Americans report getting insufficient sleep (58). However, it is important to bear in mind that previous research has demonstrated that nearly one-quarter of young adults in Australia report experiencing sleep problems and over half report sleepiness or fatigue most days (59). Therefore, it may be important to consider other aspects of sleep, such as quality and sleep patterns, in future studies as well as extending the assessment of sleep duration to older aged cohorts where this disturbance generally hits its peak (e.g., 20- to 24-year olds).

Consistent with previous research examining multiple risk behaviors among emerging adults (21–26), the present study found risk behaviors to cluster among our sample in meaningful risk profiles. Specifically, the LCA resulted in three distinct classes. Class 1 (“moderate risk”) was the most favorable group, yet was still characterized by a high probability of insufficient vegetable intake and a moderate probability of binge drinking and poor fruit consumption. Class 2 (“inactive, non-smokers”) was differentiated from the other two classes by high probabilities of physical inactivity and sitting time, and a low probability of smoking. The third class (“smokers and binge drinkers”) was characterized by high probabilities of smoking, binge drinking, and not consuming sufficient vegetables and fruit. The patterns of clustering observed in the present study are consistent with findings from previous studies in this field. For example, a recent systematic review found the strongest evidence for clustering of smoking and alcohol use among a general adult population (20), and in another review more than half of the studies reported a clustering of alcohol with smoking (60). The clustering of smoking and risky alcohol use in our study is also consistent with patterns observed in national survey data in Australia (61) and in previous samples of emerging adults (62). In addition, activity-related behaviors, such as physical inactivity and sedentary behavior, have been found to cluster with dietary behaviors, such as inadequate fruit and vegetable consumption (63–65).

The present findings have important implications in terms of prevention and early intervention. Ideally, chronic disease prevention should aim to deter risk factors from emerging in the first place (i.e., maintain good levels of sleep in the current sample), as well as reducing existing risk factors/increasing health-protective behaviors (e.g., improving vegetable consumption) (13). To achieve this, a combination of universal (i.e., delivered to an entire population) and targeted (i.e., for at-risk individuals) prevention approaches are likely to be necessary. The high prevalence of many of the six risk behaviors among the 18-year olds assessed in this study reinforces the notion that unhealthy behaviors are well-established by emerging adulthood and points to the need for prevention approaches to be implemented early in life. Preventive interventions delivered in adolescence, prior to the escalation of many risk behaviors offer an opportunity to equip young people with the capacity to make healthy decisions, increase adherence to national health guidelines, and reduce the risk of later chronic disease. For example, school is an ideal setting for intervention delivery, as educators can reach large youth audiences prior to risk behaviors becoming entrenched, and education about nutrition, physical activity, alcohol and smoking is typically included in the school curriculum (66).

Previous clustering analyses have typically identified a “healthy” class of participants, characterized by a low prevalence or absence of all risk behaviors. In fact, in a recent systematic review of the clustering of health behaviors, 81% of studies found a “low-risk/healthy” cluster (60). Although a “healthy class” could have emerged if we had sufficient data to estimate more clusters, this pattern of results was not observed in the present study, with all three classes characterized by a moderate to high probability of engaging in more than one risk behavior. For example, poor vegetable intake co-occurred with other risk behaviors in all three classes. This finding, coupled with the high prevalence of many of the six risk behaviors in the overall sample, provides support for the implementation of universal multiple health behavior prevention approaches (67), in which multiple risk factors are targeted concurrently rather than in isolation. This approach capitalizes on evidence that modifying one risk behavior can lead to improvement in another (68). For example, research has shown that targeting fruit/vegetable consumption and sedentary leisure time together can lead to untargeted reductions in fat intake in adults (69). This is often referred to as the “transfer effect” (70–72), whereby the lessons, skills, and knowledge learned in one behavioral context are applied to another context, assuming an individual has the capacity to apply acquired competences to other domains (73). An understanding of the combinations of risk behaviors that young people commonly engage in has important implications for the development and tailoring of universal multiple health behavior interventions. Previous research suggests that intervening synergistically via multiple health behavior interventions offers a potentially efficient (74) and cost-effective means (24) of educating young people about key risk factors for chronic disease, however, further research is needed.

Our findings also point toward the implementation of targeted prevention and early intervention approaches. In the current study, three distinct classes were identified, with some classes reporting very high rates of a behavior (e.g., smoking in Class 3) and others reporting very low rates (e.g., non-smoking in Class 2). The identification of young people based on their unique risk factor profiles, and subsequent tailoring of interventions, may improve engagement with, and efficacy of, multiple health behavior programs (75, 76). In particular, self-monitoring and tracking of behaviors (e.g., diet, activity, and sleep) *via* mobile applications and online interventions offer a potentially engaging and effective way for individuals to address behaviors for which they have been deemed at-risk (77). The use of mobile technologies has been shown to be effective in improving physical activity and smoking cessation among adults (78) and there is increasing evidence to support the use of smartphone applications to improve health behaviors in youth (79–81).

Although Class 2 (“inactive, non-smokers”) had significantly lower levels of employment than the other two classes, there were no differences in terms of education and overweight/obesity (mean BMI within normal range for all classes), and gender differences between the classes were not significant when taking into account uncertainty in class assignments. Findings from the present study replicate previous work which has demonstrated associations between multiple lifestyle risk behaviors and mental health outcomes (22, 23, 29). The present results indicate that mental health symptoms were significantly greater among the two classes that exhibited high probabilities of engaging in multiple lifestyle risk behaviors. Specifically, participants in Class 3 (“smokers and binge drinkers”), which was characterized by high probabilities of smoking, drinking, and having a poor diet, had greater levels of psychological distress, anxiety, and depression, compared with Class 1 (“moderate risk”). In addition, Class 2 (“inactive, non-smokers”), characterized by high probabilities of physical inactivity, sedentary behavior, and poor diet, reported greater depressive symptoms than Class 1.

Indeed, Class 1 appears to be the “healthiest” group in the present study, exhibiting the lowest probabilities across the majority of risk behaviors, and therefore it is perhaps not surprising that this group of participants also exhibited the lowest levels of mental health symptoms. The comorbidity of alcohol and other drug use with anxiety and depression is well-established (82), and there is also evidence to support an association between poor diet and mental health problems (36, 83). The lifestyle risk profile of Class 2, physically inactive and sedentary, might reflect some overlap with depressive symptoms (e.g., fatigue, low energy, and loss of pleasure in usual activities). However, as this study only utilized cross-sectional data, no conclusions about the causality of associations between the latent classes and mental health outcomes can be determined. Previous research suggests that addressing lifestyle risk behaviors, such as diet, could lead to improvements in mental health symptoms (83, 84). Future research should examine whether interventions that jointly targeting chronic disease risk behaviors and mental health problems, before their onset, can reduce chronic disease risk and improve current physical and mental wellbeing.

### Limitations and Future Directions

This study employed sophisticated analytic methodology, LCA, and explored a wide array of risk behaviors among a sample of 18-year olds in Australia. Despite these strengths, the present results should be considered in light of a number of limitations. Firstly, this study relied on self-report data for all six risk behaviors and it is possible that participants under or over reported their behavior. Objective data, including objective measures of physical activity, sitting time, and sleep *via* accelerometers, are needed to validate self-report responses. Secondly, this study focused on only one domain per behavior, for example, sedentary behavior was represented by the number of hours spent sitting, while poor sleep was assessed *via* sleep duration per day. Future research should assess additional domains of risk behaviors, such as screen time (including device-specific time, such as TV viewing, computer use), other aspects of diet (e.g., saturated fat and sugar sweetened beverages), and sleep quality and patterns (85). Thirdly, attrition analyses revealed that participants who did complete the current 5-year follow-up assessment were more likely to be male and had higher rates of binge drinking at baseline. This suggests that the prevalence of binge drinking in our sample may have been an underestimated. In addition, although participants in the present sample were not recruited *via* universities, the vast majority were engaged in tertiary education at the time of the assessment. Although the prevalence of many risk behaviors in our sample were similar to national survey estimates among 18- to 24-year olds (15), it is possible that our sample may not be representative of all Australian young adults, especially those with lower levels of education. Previous research has demonstrated that the presence of multiple risk behaviors is higher among those with lower levels of education and high socio-economic disadvantage (20, 60, 61). Therefore, future research should seek to explore the clustering of multiple risk behaviors among young adults from a broader range of socio-economic backgrounds, and should also assess additional factors such as income and occupation. In terms of model fit, it should be noted that an entropy value of 0.74 means that more than 25% of our sample was not optimally classified. Finally, as discussed previously, this study was cross-sectional in nature and therefore no conclusions about causality can be determined. Data on the majority of risk behaviors analyzed in this study (physical activity, diet, sitting time, and sleep) were not collected during previous waves of assessment; however, planned future data collection among this cohort will enable longitudinal investigations to be conducted.

## Conclusion

Emerging adulthood (18–25 years) is an important developmental period where several risk behaviors emerge and become entrenched. Results from this study indicate that lifestyle risk behaviors are prevalent among emerging Australian adults, and that risk behaviors co-occur with one another and are associated with different levels of mental health symptoms. An examination of the clustering of risk behaviors is important for guiding the development of interventions to prevent chronic disease, as it provides insights into which risk behaviors could be targeted together. Given that chronic disease is the leading cause of death and disability in Australia, it is critical to understand how and when to optimally intervene, to promote healthy lifestyles and reduce disease risk. Our findings reinforce the importance of delivering multiple health interventions to reduce later chronic disease risk and to improve current mental wellbeing among young people.

## Ethics Statement

This study was approved by the UNSW Sydney Human Research Ethics Committee (HC16881). All subjects gave informed consent in accordance with the Declaration of Helsinki.

## Author Contributions

KC designed the research question and led manuscript preparation. MM led the statistical analysis and write-up of results. NN, MT, and KC secured funding for the study. KC, MM, BS, FK-L, and MT interpreted the results, and read and approved the final manuscript.

## Conflict of Interest Statement

The authors declare that the research was conducted in the absence of any commercial or financial relationships that could be construed as a potential conflict of interest.
